# Predicting disease progression in high-grade glioma with neuropsychological parameters: the value of personalized longitudinal assessment

**DOI:** 10.1007/s11060-019-03249-1

**Published:** 2019-07-24

**Authors:** Elke Butterbrod, Jimme Bruijn, Meriam M. Braaksma, Geert-Jan M. Rutten, Cees C. Tijssen, Monique C. J. Hanse, Margriet M. Sitskoorn, Karin Gehring

**Affiliations:** 1 Department of Cognitive Neuropsychology, Tilburg University, Warandelaan 2, 5000 LE Tilburg, The Netherlands; 2 Department of Neurology, Elisabeth-Tweesteden Hospital, Hilvarenbeekseweg 60, 5022 GC Tilburg, The Netherlands; 3 Department of Neurosurgery, Elisabeth-Tweesteden Hospital, Hilvarenbeekseweg 60, 5022 GC Tilburg, The Netherlands; 4 Department of Neurology, Catharina Hospital, Michelangelolaan 2, 5623 EJ Eindhoven, The Netherlands

**Keywords:** Cognitive functioning, High-grade glioma, RANO, Disease progression, Neuropsychological assessment

## Abstract

**Purpose:**

Progressive disease in patients with high-grade glioma may be reflected in cognitive decline. However, the cognitive functions most sensitive to progression may differ between patients. We investigated whether decline on a personalized selection of tests predicted progressive disease according to RANO criteria in high-grade glioma patients.

**Methods:**

Starting one day before surgery, patients underwent neuropsychological assessment every three months during standard treatment and clinical follow-up. We first made a personalized selection of three tests that showed the highest Reliable Change Index (RCI) values, i.e., most positive change, at the first post-surgical assessment for each patient. In subsequent follow up, a decline of RCI ≤ − 1 on at least two of the three tests in the selection was considered cognitive decline. We performed a discrete Cox proportional hazards model including a time-dependent coefficient cognitive decline (vs. stability) and covariate age to predict progressive disease.

**Results:**

Twenty five patients were included. Cognitive decline on the personalized test selection preceded or had occurred by the time progression was established in 9/15 patients with RANO confirmed progressive disease (60%). Decline was absent in 8/10 patients (80%) with stable disease during participation. The independent hazard ratio for progression in case of cognitive decline was 5.05 (*p* < 0.01) compared to stable performance.

**Conclusions:**

Using only three patient-specific neuropsychological tests, we found a fivefold increased chance of disease progression in case of cognitive decline as compared to stable performance. Brief, patient-tailored cognitive assessment may be a noninvasive addition to disease monitoring without overburdening patients and clinical care.

**Electronic supplementary material:**

The online version of this article (10.1007/s11060-019-03249-1) contains supplementary material, which is available to authorized users.

## Introduction

Identification of reliable prognostic indicators for disease progression and overall survival is a principal aim in care for patients with high-grade gliomas (HGG), for treatment planning and to inform patients. Age and performance status (PS) are generally considered the major prognostic factors from a clinical perspective [1–3].These characteristics, however, may be interrelated with or confounded by other factors, such as therapeutic strategy or varying disease symptoms [1, 4], that are not always accounted for.

Interest in the prognostic value of cognitive functioning in the clinical management of glioma patients is growing [1, 5–9]. Cognitive functioning depends on neuronal synchrony across brain regions [10]. Invasive growth, reactive changes in peritumoral tissue and increased intracranial pressure may all disrupt network functioning needed for cognitive performance [11].

Cognitive status before surgery or oncological treatment has been reported as a predictor of (progression-free) survival time [7] independent of age and Karnofsky PS (KPS) [9] and within RPA-RTOG classes [12]. Furthermore, Meyers [8] reported that performance decline over time on one of nine cognitive tests preceded radiological evidence of progressive disease (PD) in HGG in 85% of cases and by a median of 4 to 7 weeks. In a heterogeneous sample of patients with brain tumors, Armstrong and colleagues [5] showed that a decline of one standard deviation (SD) on the standardized mean of three to five tests per patient selected based on tumor location, was accompanied by a fivefold increase in chance of PD.

Cognitive deterioration over time might thus provide information about tumor activity during the course of the disease [5, 8, 13, 14]. A targeted test selection, e.g., tumor location-based as done by Armstrong [5] may increase efficiency of assessment. However, dysfunction of specific cognitive domains may not be reliably determined by location alone [15, 16] as tumors affect cerebral functioning outside their location [17].

In this study, we argue that disease-related cognitive dysfunction in HGGs, and individual differences therein, may also be detected by considering the manner in which a patient’s performance changes early after tumor resection. We hypothesize that the functions that show the largest recovery shortly after surgery are the ones that suffered the largest burden from tumor-related edema and mass effect, and that these same functions may deteriorate first amid recurrent disease activity. In a sample of newly diagnosed HGG, we constructed a personalized test selection for each patient, based on a subset of three neuropsychological tests that demonstrated most improvement within three months after resection. We subsequently investigated whether deterioration on this selection coincided with, and predicted PD.

## Materials and methods

### Patients

Patients undergoing resection at the Elisabeth-TweeSteden Hospital (ETH), Tilburg, the Netherlands, between August 2015 and September 2017 for histopathologically confirmed grade III AA or GBM were included. Patients received clinical follow up either at ETH or Catharina Hospital (CH) in Eindhoven, the Netherlands. Exclusion criteria were age < 18, presence of progressive neurological disease, psychiatric or acute neurological disorder within the past 2 years, previous intracranial surgery, reduced testability (e.g. lack of proficiency in Dutch, estimated IQ < 85). All participants provided written informed consent.

### Study procedure and design

At the neurosurgery department of ETH, patients with brain tumors undergo neuropsychological assessment (NPA) as part of clinical care 1 day before (T0) and 3 months after (T3) neurosurgical treatment. Around T3, patients were asked to participate in this prospective longitudinal study by a neuro-oncology nurse practitioner, and underwent three monthly NPA and MRI, for up to 24 months after surgery (T24; 9 NPA’s in total) or until confirmed progressive disease (PD) at their clinical follow-up site. NPA and MRI were performed on the same day, but NPA was always done before the patient was informed of the results of the MRI. Approval for the study was given by Medical Ethics Committee Brabant (File No. NL41351.008.12).

### Measures

#### Sociodemographic and clinical measures

Sociodemographic information was gathered through semi-structured interview at T0. Clinical information (tumor characteristics, extent of resection, KPS, medication, adjuvant treatment) was retrieved from electronic charts. Pre-surgical tumor volumes were determined through semi-automatic segmentation using BrainLab Elements [18] software on T1-post contrast enhanced series.

#### Neuropsychological assessment

The Dutch translation of the CNS Vital Signs (CNS VS) computerized test battery consists of seven tasks based on conventional paper-and-pencil tests [19]. Completion using the local software application on a notebook computer took 30–40 min. Two additional paper-and-pencil tasks were administered: Digit Span task [20], and a Letter Fluency task [21]. An overview of task content and score computation is provided in the supplementary Table. Trained test administrators conducted assessments.

#### MRI-cerebrum and time until PD

Evaluations of the three monthly MRI scans were conducted by a trained neurologist (MB) under supervision of a senior neurologist (CT), both unaware of patients’ cognitive status. The baseline for comparison follow up MRI scans (at T3, T6, etc.) was the first post-operative scan ( ≤ 48 h after surgery). We adopted the response assessment criteria for HGG by the RANO Working Group [22] for disease status evaluation: (1) ≥ 25% increase of the product of the maximum diameters of contrast-enhancing lesions (2) significant increase of lesions in T2-weighted/ FLAIR series (3) presence of new contrast-enhancing lesions outside radiation field (4) significant clinical deterioration not attributable to medication or comorbid conditions, or (5) clear progression of a non-measurable lesion.

### Cognitive change as a personalized predictor

#### Reliable change

Regression-based Reliable Change Indices (RCI), aimed at determining whether change between assessments in individual patients reflected relevant change, controlling for confounding factors related to repeated testing (e.g. flawed test–retest reliability, practice effects) [23, 24], were computed for each of the 10 test scores. A positive RCI value indicates improvement, a negative RCI value indicates decline. RCI’s were based on repeated testing data of healthy Dutch individuals from Rijnen [24] (CNS VS), Schmand [21] (Letter Fluency), and an ongoing study in the ETH (Digit Span test); CAR study A, ClinicalTrials.gov reference nr. NCT02953756.

#### Personalized selection and criterion for cognitive decline (CD)

For each patient, the three tests with the highest RCIs between T0 and T3 were selected. We opted to select three tests in accordance with previous similar studies [5, 8], and with the goal of a small selection of tests for potential future clinical purposes. All follow-up RCI’s were calculated using T3 NPA as baseline (T6–T3, T9–T3, etc.). CD was defined as RCI ≤ − 1.00, reflecting a standardized difference score of − 1, on at least two of the three selected tests at any follow up interval.

### Statistical analyses

Using the Survival package in Rstudio, a discrete Cox proportional hazards model with two covariates was performed (α = 0.05): a dichotomous time-dependent covariate (CD vs. stable performance) and age at time of surgery. Cases who dropped out before PD, completed follow up (T24) progression-free, or showed stable disease at the end of the study (August 2018), were censored. Median time to PD and to CD were computed. Z-scores, corrected for age, sex and educational level based on a healthy control sample [25] were computed to investigate whether patients had cognitive impairment before surgery (Z ≤ − 1.5). Group-level characteristics of patients with PD, without PD, with CD, and without CD were computed (no statistical comparisons due to sample sizes).

## Results

### Patients

Thirty-five of 70 (50%) patients eligible for participation were included in the study. Unwillingness, anticipated intensity of repeated NPA, and follow-up care in a non-participating center were reasons for declining participation. Ten out of 35 patients were excluded from analyses, because of invalid or incomplete T0 NPA (*n* = 6), or absent T6 data (consent withdrawal; *n* = 3, referral to non-participating treatment center; *n* = 1). Analyses showed no differences in age, tumor volume, extent of resection or pre-operative KPS between excluded patients and the final sample.

The final sample comprised four anaplastic astrocytoma (AA) and 21 glioblastoma (GBM). Mean age at time of surgery was 53 ± 14 years. See Table 1 for an overview of sample characteristics and Table 2 for the personalized test selection per patient. All patients started adjuvant chemoradiation according to protocol [26]. Twenty-one patients completed treatment as planned during study participation. Temozolomide monotherapy was discontinued in four patients, either on patient’s request (*n* = 1, around T6), because of PD during (*n* = 2, around T6) or due to treatment-related toxicity (*n* = 1, between T6 and T9).

**Table 1:** Sample characteristics

Characteristic	N = 25
Male *n* (%)	17 (68%)
Age at time of surgery (M ± SD, range)	53 ± 14, 19–76
Educational level^a^	
Low *n* (%)	3 (12%)
Middle *n* (%)	12 (48%)
High *n* (%)	10 (40%)
Diagnosis	
Glioblastoma	21 (84%)
Anaplastic astrocytoma	4 (16%)
Tumor volume (cm^3^), median (range)	37 (4.4–162)
KPS before surgery, mode (range)	90, 80–100
Tumor lateralization *n* (%)	
Right	15 (60%)
Left	10 (40%)
Tumor location *n* (%)	
Frontal	6 (24%)
Fronto–parietal	1 (4%)
Parietal	3 (12%)
Parieto–temporal	2 (8%)
Parieto–occipital	4 (16%)
Temporal	3 (12%)
Occipital	6 (24%)
Corticosteroids before surgery	17 (68%)
Anti-epileptics before surgery	8 (32%)
Macroscopic extent of resection	
Gross total resection ( > 90%)	17 (68%)
Gross subtotal resection ( < 90%)	8 (31%)

^a^Classified according to Verhage education coding system [31]

**Table 2:** Cognitive parameters and tumor location per patient

Diagnosis	Age	Location	Hemisphere	Selected tests	Impairment on selected tests at T0^a^	PD during follow up
GBM	18–20	Frontal	Left	SAT, LF, SDC	3/3	No^c^
GBM	60–70	Parieto–occipital	Right	SAT, VEM, SDC	2/3	Yes
AA	30–40	Frontal	Left	DSFW, LF, SAT	1/3	No^c^
GBM	60–70	Occipital	Left	VIM, DSB, DSF	0/3	Yes
GBM	50–60	Occipital	Right	SDC, CPT, FTT	2/3	Yes
GBM	50–60	Fronto–parietal	Right	SAT, VIM, VEM	1/3	Yes
GBM	40–50	Frontal	Right	SAT, CPT, FTT	2/3	Yes
GBM	60–70	Parietal	Right	CPT, SAT, VEM	3/3	Yes
GBM	60–70	Occipital	Right	VIM, SAT, VEM	3/3	Yes
GBM	40–50	Frontal	Left	FTT, CPT, SAT	0/3	Yes
GBM	50–60	Parieto–occipital	Left	SAT, CPT, SDC	1/3	No^b^
GBM	50–60	Parietal	Left	SAT, VIM, FTT	3/3	Yes
GBM	50–60	Parieto–occipital	Right	FTT, CPT, SDC	3/3	No^b^
AA	30–40	Frontal	Right	VIM, VEM, LF	0/3	No^c^
GBM	60–70	Temporo–parietal	Left	FTT, SAT, Stroop	0/3	Yes
GBM	70–80	Parietal	Right	FTT, SAT, Stroop	2/3	Yes
GBM	50–60	Frontal	Right	FTT, VIM, DSF	0/3	Yes
GBM	50–60	Occipital	Right	VEM, VIM, LF	2/3	Yes
GBM	50–60	Temporo–parietal	Left	FTT, VEM, Stroop	0/3	Yes
GBM	30–40	Occipital	Right	FTT, DSF, SDC	2/3	No^b^
GBM	70–80	Temporal	Right	Stroop, SAT, VIM	0/3	Yes
GBM	50–60	Occipital	Right	VIM, Stroop, FTT	1/3	No^a^
GBM	50–60	Mesiotemporal	Left	FTT, LF, SDC	3/3	No^a^
AA	50–60	Temporal	Right	SAT, SDC, DSB	3/3	No^a^
AA	20–30	Parieto–occipital	Left	FTT, VEM, DSB	2/3	No^a^

^a^Active participation at end of study

^b^Dropout before PD

^c^Completion of T24 without PD

In 23 out of 25 patients, all three selected tests with the highest RCI from T0 to T3 were positive scores ( > 0), indicating improvement after surgery. In two patients, the selection also contained tests with negative values (highest RCI’s were 0.62, 0.23, and − 0.25 in one patient, 1.76, − 0.18, and − 0.20 in another patient).

Fifteen out of 25 patients (60% of the sample) showed PD according to RANO during follow up (see Table 3 for evaluations of PD). Eleven out of 25 showed CD during follow up. Within the “PD group”, the median time-point to PD was T9, while within the “CD group”, the median time-point to CD was T6.

**Table 3:** Descriptive characteristics and RANO [22] evaluations grouped by disease status and cognitive status on personalized test selections

Progressive disease (n = 15)	Decline on tests (n = 9)	Stable on tests (n = 6)
Age before surgery	60.0 ± 7.6	58.3 ± 10.7
Low education	0 (0%)	1 (16.7%)
High education	4 (44.4%)	3 (50%)
Male	7 (77.8%)	5 (83.3%)
Impairment on ≥ 1 selected test at T0	5 (55.6%)	5 (83.3%)
Tumor in left hemisphere	4 (44.4%)	1 (16,7%)
Macroscopic total resection	6 (66.7%)	3 (50%)
Time to CD (median)	T6	n/a
Time to PD (median)	T6	T12
KPS < 90 at time of PD	5 (55.6%)	0 (0%)
AEDs at time of PD (%, n at T3)	6 (66.7%, 4)	1 (16.7%, 1)
Corticosteroids at time of PD	2 (22.2%)	0 (0%)
RANO evaluation		
New contrast-enhancing lesion outside radiation field^a^	0 (0%)	1 (16.7%)
Increase ≥ 25% in the sum of the products of perpendicular diameters	5 (55.6%)	3 (50%)
Clinical deterioration not attributable to medication or comorbidity ( ≥ 12 weeks post-chemoradiation)	1 (11.1%)	0 (0%)
Significant increase in T2/FLAIR non-enhancing lesion	0 (0%)	0 (0%)
Clear progression of a nonmeasurable lesion	3 (33.3%)	2 (33.3%)

Stable disease (n = 10)	Decline on tests (n = 2)	Stable on tests (n = 8)
Age before surgery	55.5 ± 3.54	40.3 ± 15.6
Low education	0 (0%)	2 (25%)
High education	1 (50%)	4 (50%)
Male	2 (100%)	3 (37.5%)
Impairment on ≥ 1 selected test at T0	2 (100%)	7 (87.5%)
Tumor in left hemisphere	1 (50%)	4 (50%)
Macroscopic total resection	2 (100%)	6 (75%)
Time to CD (Median)	T6	n/a
Time to PD (Median)	n/a	n/a
KPS < 90 at time of censoring	0 (0%)	0 (0%)
AEDs at time of censoring (%, n at T3)	0 (0%, 0)	2 (25%, 2)
Corticosteroids at time of censoring	0 (0%)	0 (0%)

Percentages are calculated within each group

^a^Only case of non-local tumor recurrence

**Fig. 1:**
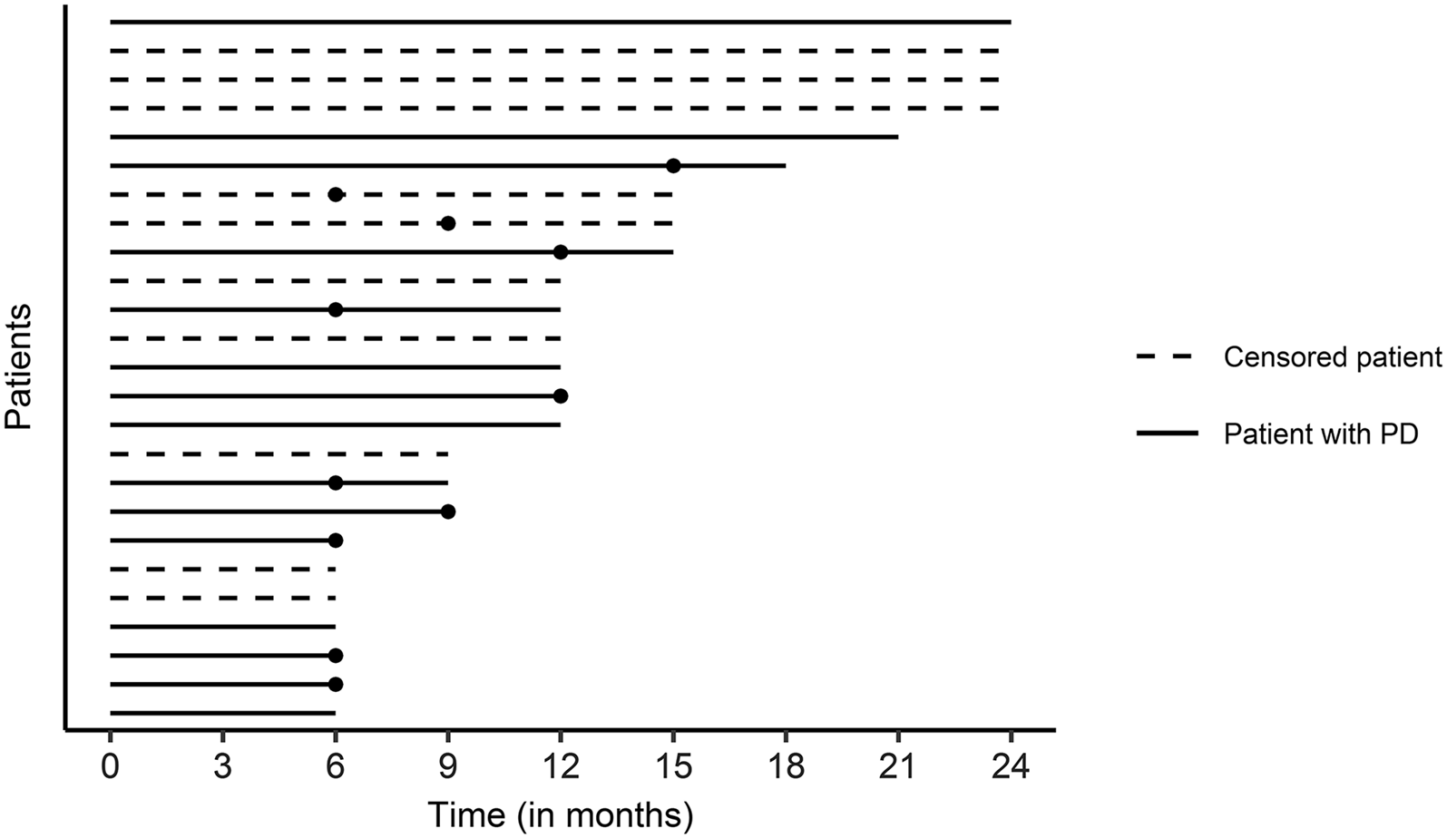
Follow-up duration per patient and time of CD (filled circle) and PD. Lines stop at time of RANO PD (bold line) or end of participation (dotted line; censoring). Timing of CD differed between patients (one patient two intervals before PD, two patients one interval before PD, five patients at time of PD)

CD preceded (n = 4) or was present at time of (n = 5) PD in nine out of 15 patients with PD. See Fig. 1 for a visualization of individual follow-up periods and the timing of CD relative to PD. Five of the six patients who showed stable cognitive performance according to our criterion, despite PD, showed RCI ≤ − 1 on one of their selected tests at time of PD, while showing no RCI ≤ − 1 on their unselected tests.

In 10 of 25 patients (40%), PD did not occur during study participation. Eight out ten (80%) were stable performers throughout follow up (median follow up time-point T12, range T6–T24). Two patients showing CD despite stable disease, did so at T6 and T9 respectively.

Table 3 shows descriptive characteristics of the four groups (no statistical comparisons). The group demonstrating both CD and PD was the only group in which KPS below 90 was observed at time of final NPA. AED use was high among these patients compared to the other groups, but the majority (four out of six) used medication because of a pre-surgical insult. The other two started AED therapy due to a seizure during follow up (both in the interval prior to PD). The group with stable disease and stable cognitive performance appeared relatively young and to comprise fewer males compared to the other groups.

The Cox proportional hazards model showed a hazard ratio (HR) for PD of 5.05; 95% CI 1.50–17.02, *p* < 0.01 (model χ^2^[1] = 13.6, *p* < 0.01, c-index = 0.80), suggesting a 405% increase in chance of RANO-confirmed PD if patients met the criterion of CD compared to stable cognitive performance, independent of age. Age itself was not a significant predictor (HR = 1.04, *p* > 0.1) of PD.

## Discussion

This study investigated whether post-surgical cognitive decline (CD) on a personalized selection of three neuropsychological tests concurred with and predicted progressive disease (PD) according to RANO [22] in 25 patients with GBM or AA.

Decline in cognitive performance—deterioration of at least one RCI point on at least two of the three selected tests—concurred with, or manifested one or two interval(s) before, RANO-confirmed PD in nine out of 15 (60%) of recurrences. Of the six patients with PD who did not meet our criterion for CD, five showed RCI ≤ − 1on one of their selected tests, but no such decline on their unselected tests. Further consistent with our hypothesis and previous reporting [27], eight out of 10 patients with stable disease remained cognitively stable throughout participation. The predictive model showed a 405% increase in chance for PD (HR = 5.05) in case of CD, independent of age.

Our findings support existing reports of (change in) cognitive functioning as a clinical marker of disease activity in patients with brain tumors [5, 8, 13, 28]. Gradual, widespread impairment of network functioning over time may underlie the sensitivity of cognitive performance to disease progression. Using a uniform test selection in patients with recurrent HGG, Meyers and colleagues [8] reported a higher proportion of patients showing CD (48/56 patients, CD was defined as RCI ≤ − 1.645 on one of nine tests) before, or at time of, PD compared to our study. It could be that CD emerges sooner in patients with progression of already recurred HGG. Still, the described criterion for decline was based on a more stringent cutoff, but for only one test, and time between CD and actual PD seemed to vary considerably among patients. Their reported prediction model (requiring decline on one of three uniform tests) yielded a HR for PD of 2.0 in case of CD.

The hazard ratio of CD (one standard deviation in mean performance) on a tumor location-based selection of three to five tests found by Armstrong [5] in a sample of 34 patients with glial and non-glial tumors, of which 11 demonstrated recurrence, was comparable to the one we found. As stated, reliable inferences about cognitive (dys-)function may not be based on tumor location alone [15]. In our sample of mainly GBMs, we did not observe a one-to-one relationship between tumor location and the personalized selection of tests, e.g., Letter Fluency and Shifting Attention tests were selected in patients with occipital tumors. The relative value of selection approaches (uniform, location-specific, personalized) within the context of prediction of PD may be compared in one larger sample in the future.

The absence of a gold standard concerning the cut off for CD, irrespective of the selection approach, in settings where cognition is used as a predictive instead of an outcome measure also warrants further investigation. The used RCI is a suitable measure for change as it conveys a cautious estimation of decline. Selecting three tests per patient is in accordance with previous approaches [5, 8] and preserves briefness required for repeated NPA in the HGG population. We must note that the widely adopted RANO criteria for HGG are based on current evidence [22] and will likely evolve in the future.

Cognitive performance can fluctuate over time due to factors (un-)related to disease activity, such as temporary corticosteroid use [29] or depressive symptoms [30]. It has however been suggested that the main cause of cognitive decline over time is the tumor itself [13]. Cognitive stability in eight out of 10 progression-free patients in this study also suggests that such factors did not disturb cognitive performance strongly (RCI’s remained > − 1) or in a personalized pattern. The group with both CD and PD did seem to comprise a relatively large proportion of patients using AED’s, although in the majority of cases due to a pre-surgical insult. It was also the only group that comprised patients with KPS < 90 at time of final NPA/PD. Clinical status may have interplayed with cognitive functioning around the time of PD. We were unable to adopt post-surgical decline in KPS or a combined cognition-KPS classification in the statistical model due to sample size, but analysis of hazard rates associated with CD irrespective of (K)PS decline could be a next step in future research.

Sample characteristics should be taken into account in interpreting our results. We note that our sample primarily comprised patients with GBM, and the majority of AA patients did not show PD. Moreover, 50% of patients who were invited to participate in this study declined participation, e.g., due to anticipated intensity of repeated NPA. Patients in good clinical condition at time of inclusion might be overrepresented. Furthermore, only 12% of the included patients had low educational level. Different cognitive courses might exist between groups who differ on these variables.

Results from the personalized prediction model warrant further investigation to establish relevance in clinical practice. Personalized NPA may serve as a noninvasive method to complement decision making processes, such as timing of second-line therapy in case of unclear or seemingly limited tumor growth. Conducting targeted NPA between MRI scans may also allow for early detection of recurrent disease activity, e.g., in patients whose radiological evaluation is conducted over longer intervals due to other relatively favorable prognostic features.

## Conclusion

In conclusion, our prediction model based on a personalized selection of three neuropsychological tests showed CD before or at time of PD in the majority of patients with HGG. Eighty percent of progression-free survivors showed stable cognitive performance. Patients demonstrating CD showed five times higher chance of PD compared to stable performers. Personalized, longitudinal NPA may provide a targeted and sensitive addition to monitoring of both cognitive and disease status without overburdening patients or care trajectories.

## Electronic supplementary material

Below is the link to the electronic supplementary material.

Supplementary file1 (DOCX 14 kb)
