# Supplementary material for: Predicting disease progression in high-grade glioma with neuropsychological parameters: the value of personalized longitudinal assessment
Source: J Neurooncol. 2019 Jul 24;144(3):511–8. doi: 10.1007/s11060-019-03249-1 (PMC6764928; doi:10.1007/s11060-019-03249-1)
Supplement: Supplementary file 1 — Supplementary file1 (DOCX 14 kb) [file 11060_2019_3249_MOESM1_ESM.docx]

**SUPPLEMENTARY TABLE**

**Title:** Predicting disease progression in high-grade glioma with neuropsychological parameters: the value of personalized longitudinal assessment

**Authors:** Elke Butterbrod MSc^1†^, Jimme Bruijn MSc^1†^, Meriam M. Braaksma MD^2^, Geert-Jan M. Rutten MD PhD^3^, Cees C. Tijssen MD PhD^2^, Monique C.J. Hanse MD^4^, Margriet M. Sitskoorn PhD^1^ & Gehring Karin PhD^1,3^

^1^ Tilburg University, Department of Cognitive Neuropsychology, Warandelaan 2, 5000 LE Tilburg, The Netherlands

^2^ Elisabeth-Tweesteden Hospital, Department of Neurology, Hilvarenbeekseweg 60, 5022 GC Tilburg, The Netherlands

^3^ Elisabeth-Tweesteden Hospital, Department of Neurosurgery, Hilvarenbeekseweg 60, 5022 GC Tilburg, The Netherlands

^4^ Catharina Hospital, Department of Neurology, Michelangelolaan 2, 5623 EJ Eindhoven, The Netherlands

† joint first authorship

**Corresponding author:** Elke Butterbrod, [E.Butterbrod@tilburguniversity.edu](mailto:E.Butterbrod@tilburguniversity.edu), tel: +31 13 466 8968

| **Supplementary Table.** Description of neuropsychological tests | | |
| --- | --- | --- |
| Test | Content | Scores and computation |
| CNS VS Verbal Memory (VEM) Test | Fifteen words are presented, one at a time. Subject subsequently identifies presented words among new words. | Total items correct |
| CNS VS Visual Memory (VIM) Test | Fifteen abstract images are presented, one at a time. Subject subsequently identifies presented images among new images. | Total items correct |
| CNS VS Finger Tapping Test (FTT): Motor speed | Subject presses space bar as quickly as possible for 10 secs (index finger, three trials per side). | Taps right average + taps left average |
| CNS VS Symbol Digit Coding (SDC) Test: Psychomotor speed | Participant matches numbers with corresponding symbols for two minutes. | Correct responses – incorrect responses |
| CNS VS Stroop Test: Interference | Part 1: subject presses space bar when a word is presented.  Part 3: subject presses space bar if the color of the word does not match the meaning of the word. | (Reaction time Part 3 – Reaction time Part 1) / Reaction time Part 1 |
| CNS VS Shifting Attention Test (SAT): Cognitive flexibility | Subject matches geometric objects by shape or color for two minutes. | Correct responses – Errors |
| CNS VS Continuous Performance Test (CPT): Vigilance | Subject responds to target letter among distractors for 5 minutes. | Average reaction time of responses to B |
| Digit Span Forward (DSFW)^a^: Attention | Subject repeats series of digits of increasing length. | Total items correct |
| Digit Span Backward (DSBW) a: Acoustic working memory | Subject repeats series of digits of increasing length in reverse order | Total items correct |
| Letter Fluency^a^: verbal (lexical) ability, executive control | Subject names words starting with a specific letter for 1 minute (3 trials total). Three alternate test forms were used. | Total words correct |
| † paper-and-pencil test | | |
